# Associations of Serum Levels of Sex Hormones in Follicular and Luteal Phases of the Menstrual Cycle with Breast Tissue Characteristics in Young Women

**DOI:** 10.1371/journal.pone.0163865

**Published:** 2016-10-07

**Authors:** Linda Linton, Monica Taylor, Sheila Dunn, Lisa Martin, Sonia Chavez, Greg Stanitz, Ella Huszti, Salomon Minkin, Norman Boyd

**Affiliations:** 1 Campbell Family Institute for Breast Cancer Research, Toronto, ON, Canada; 2 Sunnybrook Health Sciences Centre, Toronto, ON, Canada; 3 Princess Margaret Cancer Centre, and Imaging Research, Toronto, ON, Canada; 4 Family Practice Health Centre, Women’s College Hospital, Toronto, ON, Canada; University of Arkansas for Medical Sciences, UNITED STATES

## Abstract

**Background:**

In previous work in young women aged 15–30 years we measured breast water and fat using MR and obtained blood for hormone assays on the same day in the follicular phase of the menstrual cycle. Only serum growth hormone levels and sex hormone binding globulin (SHBG) were significantly associated with percent breast water after adjustment for covariates. The sex hormones estradiol, progesterone and testosterone were not associated with percent water in the breast in the follicular phase of the menstrual cycle. In the present study we have examined the association of percent breast water with serum levels of sex hormones in both follicular and luteal phase of the menstrual cycle.

**Methods:**

In 315 healthy white Caucasian young women aged 15–30 with regular menstrual cycles who had not used oral contraceptives or other hormones in the previous 6 months, we used MR to determine percent breast water, and obtained blood samples for hormone assays within 10 days of the onset of the most recent menstrual cycle (follicular phase) of the cycle on the same day as the MR scan, and a second blood sample on days 19–24 of the cycle. Serum progesterone levels of > = 5 mmol/L in days 19–24 were used to define the 225 subjects with ovulatory menstrual cycles, whose data are the subject of the analyses shown here.

**Results:**

SHBG was positively associated with percent water in both follicular and luteal phases of the menstrual cycle. Total and free estradiol and total and free testosterone were not associated with percent water in the follicular phase, but in young women with ovulatory cycles, were all negatively associated with percent water in the luteal phase.

**Conclusions:**

Our results from young women aged 15–30 years add to the evidence that the extent of fibroglandular tissue in the breast that is reflected in both mammographic density and breast water is associated positively with higher serum levels of SHBG, but not with higher levels of sex hormones.

## Introduction

Mammographic density is a heritable quantitative trait that is a strong risk factor for breast cancer in middle aged and older women[1, 2]. Little is known about the factors that influence this risk factor in early life. To avoid use of radiation we have used magnetic resonance (MR) to measure the water content of the breast, which like mammographic density, reflects fibro-glandular breast tissue [3].

Several established risk factors for breast cancer, including age at menarche, age at first pregnancy, parity, age at menopause, postmenopausal obesity, and use of combined hormone replacement therapy, suggest that variations in exposure to estrogen play a role in the etiology of the disease[4]. Meta-analyses have shown serum levels of estradiol to be associated with risk of breast cancer in both premenopausal [5] and postmenopausal women [6]. Some menstrual and reproductive risk factors for breast cancer, notably parity and menopause and combined hormone therapy, but not estrogen alone, also influence percent mammographic density (PMD) [7, 8]. These data suggest that estrogen, or other sex hormones, may also influence PMD.

In previous work in young women aged 15–30 years we measured breast water and fat using MR and obtained blood for hormone assays on the same day in the follicular phase of the menstrual cycle [9]. Only serum growth hormone (GH) levels and sex hormone binding globulin (SHBG) were significantly and positively associated with percent breast water after adjustment for covariates. The sex hormones estradiol, progesterone and testosterone were not associated with percent water in the breast in the follicular phase of the menstrual cycle.

In the present study we have examined the association of these sex hormones, SHBG and prolactin measured in the luteal phase of the menstrual cycle with percent breast water, and compare the results obtained in follicular and luteal phases. Anovulatory menstrual cycles are common in young women and we have analyzed the results according to the luteal phase defined by the serum progesterone level.

## Methods

### A. General method

Between 2003 and 2006 we recruited healthy white Caucasian young women aged 15–30, and from 2007 to the present young women aged 15–18. Written informed consent from both subjects and their mothers was required before participation. These procedures were approved by ethics committees of the University Health Network, Sunnybrook Health Sciences Centre, Mount Sinai Hospital, Toronto, The Toronto District School Board and The Toronto District Catholic School Board. We used MR to examine breast tissue and also obtained anthropometric measures and other data that were used to examine factors associated with variations in breast tissue, including blood samples for hormone assays. With the consent of these school boards, and several private schools, we have presented information about the study in Toronto high schools and invited eligible young women to participate. To date a total of 957 young women aged 15–18 and their mothers have given written informed consent to take part in the study.

A subset of 315 of these young women, who had regular menstrual cycles and had not used oral contraceptives or other hormones during the previous 6 months were asked to take part in the present study and provide the measurements and data described below.

### B. Measurements

#### 1. MR measures of breast water and fat

The first 181 young women included in the analysis were examined using a 1.5T Signa Cvi MR system (GE, Waukesha WI) using previously described methods [9]. The remaining 44 subjects were examined in a 3.0T scanner (Phillips). All scans were carried out in the prone position with commercially available breast coils from the respective vendors.

To determine if the data from these two scanners could be pooled we scanned 12 healthy volunteers on both scanners on the same day. Total fat and water measures from the two scanners were strongly correlated (R^2^>0.99) and showed some overestimation of fat in the 1.5 T protocol relative to the T3.0 protocol. We therefore applied a correction applied to the T3.0 protocol that decreased the discrepancy [10].

With both scanners the sequence was carefully calibrated for both volume and fat/water percentages using a series of home-built phantoms. A QC program was maintained throughout this study to verify the accuracy and stability of these MR measurements. Bi-monthly scans of three phantoms with known water/oil concentrations and various volumes using the same MR imaging protocol confirmed volume accuracy within 2% and water/oil content accuracy within 3%.

The output of the MR examination was a series of “slices” at 7-mm intervals through both breasts. The breast was distinguished from surrounding tissues on each slice by an observer using a locally-developed, semi-automated image analysis program, and the water and fat within each slice calculated and summed over all slices which acquires the water and fat signals with phase shifts of (0, pi, 2pi). Results shown below are measurements in the right breast only and are expressed as percent water, and in S1–S3 Tables as total breast water and fat. We have shown percent breast water and total water and fat measurements to be bilaterally symmetrical [11].

#### 2. Data collection

We collected data by questionnaire on menstrual, reproductive and familial risk factors, and measured height and weight. We collected venous blood samples in the follicular phase of the menstrual cycle after a 12-hour fast early on the morning of the day of the MR examination, and within 10 days of the onset of the last menstrual period. We collected a second fasting blood collection in the assumed luteal phase, 19–24 days after the onset of the last menstrual period. At each collection, serum was separated within 2 hours, divided into 2 ml aliquots, and stored at –70°C until analysis.

#### 3. Hormone assays

Assays were performed in the Pathology Laboratory of Mount Sinai Hospital Research Services, Toronto, Canada. The methods used, coefficients of variation (CV) and minimum detectable levels (MDL) were as follows. All assays were immunoassays from "ECLIA", Roche Diagnostics. Estradiol: (CV: 9.1%; MDL: 18.4pmol/L);. Testosterone: (CV: 4.2%; MDL:0.069 nmol/L (0.02 ng/mL)); Progesterone: (CV: 5.8%; MDL: 0.095nmol/L); SHBG: (CV: 3.5%; MDL: 0.35 nmol/L); Prolactin: (CV: 1.9%; MDL:0.47 ng/ml). Free hormone levels were calculated according to the law of mass action described by Sodergard [12]. Coefficients of variation of <10% were considered acceptable.

In 90 subjects serum progesterone levels in the assumed luteal phase were less than 5 mmol/L and these subjects were excluded from the analysis, which was carried out in the remaining 225 subjects in whom serum progesterone levels indicated that they had ovulated. All hormone levels in the remaining 225 subjects were above the lower level of detection with the exception of eight subjects in whom prolactin was below the lower detection limit. Prolactin secretion by the pituitary is known to be episodic and these subjects were retained in the analysis.

### C. Statistical methods

Means and standard deviations were calculated for selected characteristics, and median values and interquartile ranges for hormone measurements. Hormone measurements in the follicular and luteal phase were compared using a two-sided Wilcoxon signed-rank test. Total water and total fat were log transformed for analysis.

The associations of MR breast tissue characteristics with hormone levels were examined using both univariable and multivariable regression models. The latter models included age, and age at menarche, anthropomorphic measures, and number of days since the most recent menstual period (LMP).

**Fig 1:**
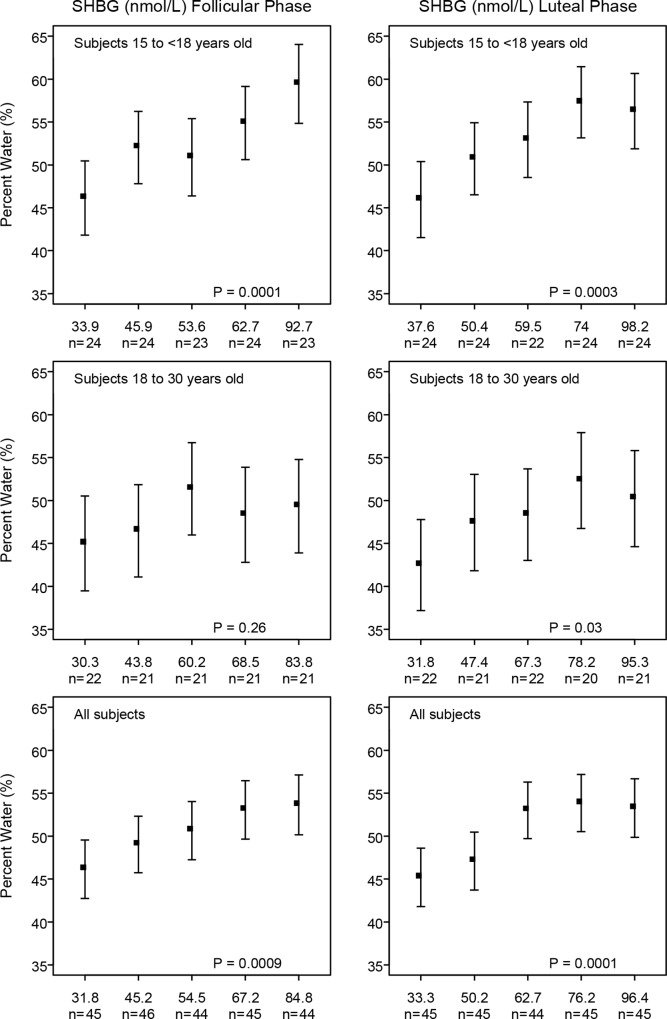
SHBG at follicular or luteal phase with MRI measurements in young women. Young women with progesterone levels less than 5 in the luteal phase were excluded. Model adjusted for *Age at MRI*; *Age at Menarche*; *Weight*; *Height* and *Days since LMP* for each phase. P: test for linear trend.

**Fig 2:**
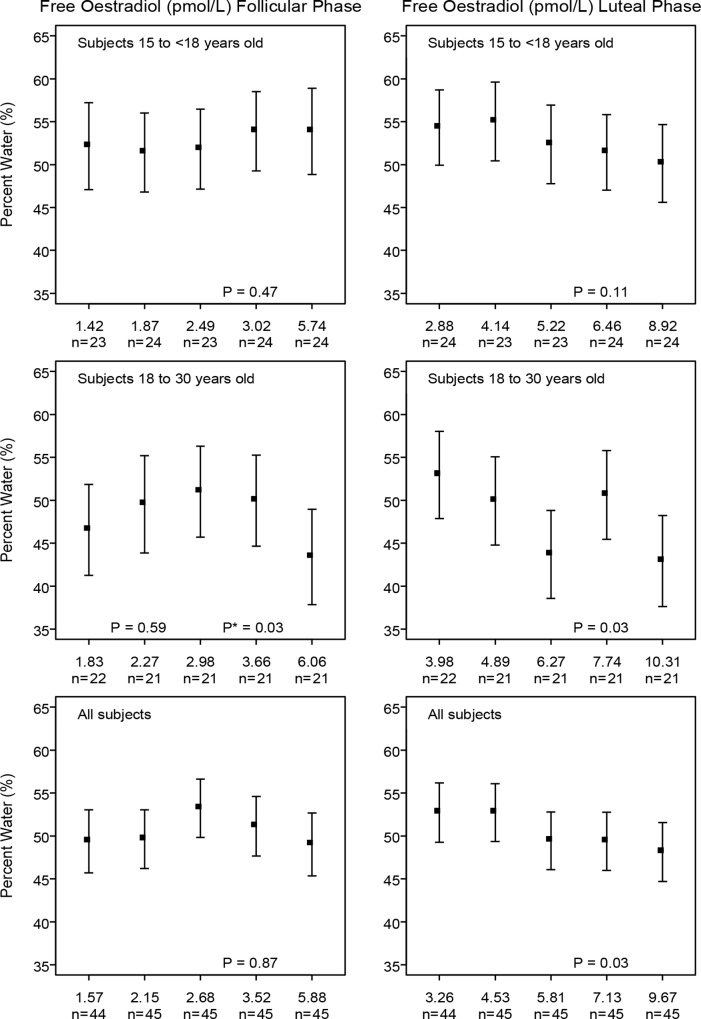
Free Estradiol at follicular or luteal phase with MRI measurements in young women. Young women with progesterone levels smaller than 5 in the luteal phase were excluded. Model adjusted for *Age at MRI*; *Age at Menarche*; *Weight*; *Height* and *Days since LMP* for each phase. P: test for linear trend; P*: test for quadratic trend

**Fig 3:**
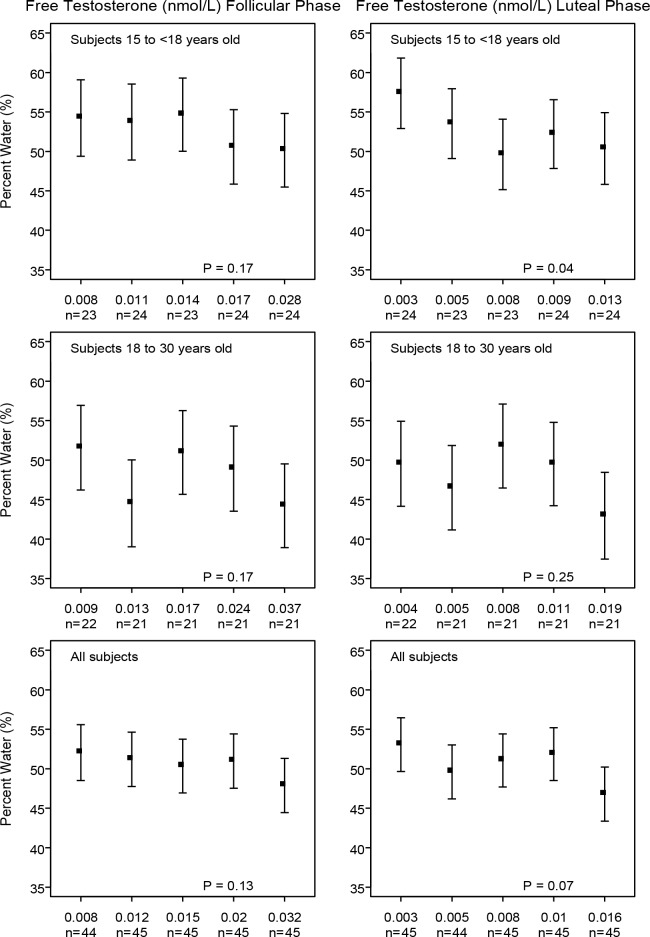
Free testosterone at follicular or luteal phase with MRI measurements in young women. Young women with progesterone levels less than 5 in the luteal phase were excluded. Model adjusted for *Age at MRI*; *Age at Menarche*; *Weight*; *Height* and *Days since LMP* for each phase. P: test for linear trend

To illustrate the main findings of regression analysis, we ran multivariable regression analyses with selected hormones divided into quintiles. The statistical significance of the differences in breast measures of percent water with increasing levels of these variables were assessed using tests for linear, and, when appropriate, for quadratic trend. A statistical test for quadratic trend was used when, on inspection of the quintile plots shown in Figs 1–3, the association of percent water with serum levels of a hormone was non-linear (e.g. see Fig 2 for free estradiol in subjects aged 15–18 years).

The analyses were conducted using R.2.15.1 statistical software. All p-values were calculated from two-tailed tests of statistical significance. Statistical significance was declared at 5% level. All p-values shown are adjusted for the variables shown in the table footnote.

## Results

### a. Characteristics of subjects

Table 1 shows selected characteristics of the 225 subjects with ovulatory menstrual cycles. Their mean age at the time of the MR scan was 19.9 years (standard deviation (SD) was 4.7 years). The mean age at menarche was 12.3 years (SD 1.1 years). Twenty three percent had previously used oral contraceptives (OCs), and all had stopped OCs at least 6 months before participating in the present study. The mean interval between the onset of the most recent menstrual period (LMP) and the date of the MR scan and follicular phase blood collection was 8 days (SD: 2.5 days). The luteal blood collection was on average 22.3 days after the onset of the LMP (SD: 4.0 days)

**Table 1:** Selected characteristics of subjects with ovulatory menstrual cycles.

	N = 225
**Risk Factors**^a^	
Age at time of MRI	19.9 (4.7)
Weight (kg) ^b^	59.5 (9.5)
Height (cm) ^b^	165.6 (5.5)
Body mass index (kg/m^2^) ^b^	21.6 (3.1)
Age at menarche (years) ^b^	12.3 (1.1)
Days since LMP–Follicular	8.0 (2.5)
Days since LMP–Luteal	22.3 (4.0)
Consumed alcohol at least once per week for 6 months or more^b^ (%)	29.0%
Smoked at least once per week for 6 months or more (% yes) ^b^	15.6%
First degree relative with cancer (% yes) ^b^	7.6%
Past use of oral contraceptives (% yes) ^b^	22.8%
**MRI measurements** ^c^	
Percent water (%)	50.9 (22.2)
Total water (cm^2^)	230.3 (120.2)
Total fat (cm^2^)	232.5 (240.3)
Total volume (cm^2^)	502.8 (313.9)

^a^ Continuous risk factors are expressed as mean (SD), binary risk factors expressed as % yes.

^b^ N = 224.

^**c**^ MRI measurements are expressed as median (IQR).

### b. Serum hormones in follicular and luteal phases of menstrual cycle

Table 2 shows serum levels of the measured hormones in all 225 subjects according to the follicular and luteal phases of the menstrual cycle. Serum levels of sex hormone binding globulin (SHBG), estradiol (total and free), and progesterone were each significantly higher in the luteal than in the follicular phase of the menstrual cycle (all p<0.0001). Testosterone (total and free) and prolactin were significantly lower in the luteal than in the follicular phase of the cycle (both p<0.0001).

**Table 2:** Hormone measurements in subjects with ovulatory menstrual cycles.

Hormones^a^	Follicular (N = 225)	Luteal (N = 225)
SHBG (nmol/L)	54.5 (27.4)	62.7 (35.5)
Estradiol (pmol/L)	187.0 (143.0)	434.0 (259.0)
Free Estradiol (pmol/L)	2.7 (1.8)	5.8 (3.2)
Progesterone (nmol/L)	2.0 (1.0)	23.0 (29.0)
Testosterone (nmol/L)	1.7 (1.1)	0.9 (0.7)
Free Testosterone (nmol/L)	0.015 (0.011)	0.008 (0.006)
Prolactin (ng/L) ^b^	22.0 (17.0)	16.0 (11.0)

^a^ Hormone measurements are expressed as median (IQR); test of significance of the difference between hormones in the follicular phase versus luteal phase was performed using a Wilcoxon signed-rank test, two-sided.

^b^ N = 217.

Similar results were seen when the analysis was restricted to the 187 subjects in whom follicular and luteal phase blood samples were obtained in the same menstrual cycle (shown in S1 Table).

### c. Percent breast water and serum hormone levels

Table 3 shows the regression coefficients and p-values that describe the association of each hormone with percent water in the breast, after adjustment for the variables shown in the footnote to Table 3.

**Table 3:** Association of hormones with percent water in the breast.

	*FOLLICULAR PHASE*		*LUTEAL PHASE*	
Hormones	*Adjusted Regression Coefficient*	*P-value*	*Adjusted Regression Coefficient*	*P-value*
**SHBG** (nmol/L)	7.90	<0.0001	8.16	<0.0001
**Estradiol** (pmol/L)	2.03	0.20	-0.99	0.61
**Free Estradiol** (pmol/L)	-0.37	0.82	-4.87	0.011
**Progesterone** (nmol/L)	0.99	0.56	1.27	0.25
**Testosterone** (nmol/L)	0.22	0.80	-1.03	0.55
**FreeTestosterone** (nmol/L)	-3.00	0.03	-3.12	0.02
**Prolactin**^a^ (ng/L)	-0.19	0.90	-0.74)	0.63

Young women with ovulatory menstrual cycles were used in the analysis (N = 224 in the multivariable models). All data were analyzed as continuous variables after log transformation (except testosterone). Estimated regression coefficients and corresponding p-values are shown after adjustment for covariates. Covariates were age at MRI (years), age at menarche (years), weight (kg), height (cm) and days since LMP for each phase. All hormone variables except testosterone were log transformed.

^a^ N = 217 (N = 216 for the multivariable model) in Follicular Phase.

Serum levels of SHBG were positively associated with percent water in both follicular and luteal phases (both p<0.0001). In both follicular and luteal phases of the cycle serum levels of total estradiol were not significantly associated with percent water (respectively p = 0.20 and p = 0.61 for total estradiol). Free estradiol was not significantly associated with percent water in the follicular phase (p = 0.82) but was inversely associated with percent water in the luteal phase (p = 0.01). Serum levels of progesterone were not significantly associated with percent breast water in either phase of the menstrual cycle (respectively p = 0.56 and p = 0.25 in follicular and luteal phases). Prolactin was not associated with percent water in either follicular or luteal phase (respectively p = 0.90 and 0.63).

Figs 1–3 show the directions and magnitudes, according to age, of those hormones that were significantly associated with percent water in Table 3.

Fig 1 shows the association of quintiles of SHBG with percent water according to age and the phase of the menstrual cycle. Statistically significant positive associations between SHBG and percent breast water were seen in follicular and luteal phases of the cycle in young women aged 15–18 years, but were weaker and significant only in the luteal phase for young women aged 18–30 years.

Fig 2 shows that the inverse association of free estradiol with percent water was statistically significant in all subjects, but an inverse linear association was significant only in 18–30 year old subjects (p = 0.03). In the follicular phase in 18–30 year old subjects a statistically significant quadratic association was seen (p = 0.03).

As shown in Fig 3, free testosterone was inversely associated with percent water in both follicular and luteal phases, but this inverse association was statistically significant only in the luteal phase in15-18 year old subjects.

### d. Total breast water and fat and serum hormone levels

The association of SHBG and free testosterone with percent water are likely to be the result of associations between these variables and total water or fat in the breast, measures from which the ratio percent water is calculated. Results of these analyses are shown in S2 and S3 Tables.

SHBG was significantly and inversely associated in both menstrual phases with the total fat volume of the breast (both p = 0.01), but was not significantly associated with total water in either phase (respectively p = 0.12 and 0.13). Free testosterone was not significantly associated with either total water or fat in the breast, but had an inverse association, of borderline significance (p = 0.10) with total water in the follicular phase and a weak positive association with total fat (p = 0.18) in the luteal phase.

## Discussion

Mammographic density in mid-life has been shown to be a strong, heritable risk factor for breast cancer [1, 2], that may be in part the combined result of genetic influences and growth and development in early life that determine breast tissue composition in adolescence [9]. Breast water has been shown to reflect fibroglandular tissue in the breast, as does mammographic density, and percent breast water and percent mammographic density are strongly correlated [13].

The present results show that in young women aged 15–30 years, after adjustment for weight, serum levels of SHGB were significantly and positively associated with percent water in the breast in both follicular and luteal phases of the menstrual cycle. A similar association of SHBG with breast tissue characteristics has been found previously in young women [9, 14], and in adult premenopausal and postmenopausal women by some [15, 16] but not all [17–20] investigators.

Although some previous studies have not identified any consistent change in serum levels of SHBG over the menstrual cycle, several studies have now reported higher levels of SHBG in the luteal phase compared to the follicular phase of the menstrual cycle, similar to the differences seen in the present study. These include studies in normal adult females [21, 22] [23] and in girls in puberty and adolescence [24] in whom SHBG levels were higher in the luteal than the follicular phase only in those with ovulatory cycles.

In the present study, total and free estradiol and testosterone were not significantly associated with percent water in the follicular phase of the menstrual cycle but in the luteal phase both total and free estradiol and testosterone were each significantly inversely associated with percent water.

SHBG transports estrogens and androgens in the blood and regulates access of these hormones to reproductive tissues whose growth and development they control (reviewed in [25]). In addition to the role of SHBG in hormone transport, it is known to be associated with several metabolic variables whose association with PMD has not yet been fully examined. For example, higher concentrations of SHBG have been associated with a lower risk cardio-metabolic profile, less adiposity and greater insulin sensitivity [26]. In female twin and sister studies it has been estimated that 56% of the variance (heritability) in serum levels of SHBG is explained by inherited variants [27, 28] and genetic variants associated with differences in mean serum levels of SHBG have been identified [29, 30]. It therefore appears likely that the association of SHBG with BD is not solely a reflection of BMI or weight, but may be related to the mechanism(s) underlying BD.

Strengths of the present study include the measurement of breast water by MR, which is quantitative, volumetric and requires little input from an observer, the standardized collection and handling of blood, and the collection of information on a wide range of potential confounding variables. Further, blood for hormone assays was collected and handled in a standardized manner at carefully defined times in the menstrual cycle.

A potential limitation of the study is that, for logistical reasons, MR scans were performed only in the follicular phase of the cycle. However, Graham et al showed little or no variation in breast water measured in repeated MR scans throughout the menstrual cycle in adult premenopausal women [31].

Our results add to the evidence that the extent of fibroglandular tissue in the breast that is reflected in both mammographic density and breast water is not associated with higher serum levels of sex hormones in either the follicular or luteal phases of the menstrual cycle. Few studies of blood levels of ovarian hormones and PMD in premenopausal women have been published to date and none have found an association [32–35].[36, 37]

Further, circulating sex steroid levels and mammographic density have been found to be strongly and independently associated with the risk of breast cancer in postmenopausal women in 2 distinct populations. While both mammographic density and serum levels of sex hormones show evidence of heritability, there is no evidence of overlap between them [38] and no evidence of a shared genetic basis between any sex hormone and any mammographic measure. However, percent mammographic density (PMD) has been shown to increase after use of combined hormone therapy, although not with estrogen alone [7], and to be reduced by Tamoxifen [39].

A separate larger study now in progress will examine the association of genetic variants with breast tissue characteristics and serum levels of hormones in young women.

## Supporting Information

S1 Table Hormone measurements in follicular and luteal phases of the same menstrual cycle. (DOC)

S2 Table Simple and Multiple regression analysis of Water Volume^a^ (MRI) in young women (ages 15–30). N = 225.
(DOC)

S3 Table Simple and Multiple regression analysis of Fat Volume ^a^ (MRI) in young women (ages 15–30). N = 225.
(DOC)
