# Supplementary material for: Associations of Serum Levels of Sex Hormones in Follicular and Luteal Phases of the Menstrual Cycle with Breast Tissue Characteristics in Young Women
Source: PLoS One. 2016 Oct 7;11(10):e0163865. doi: 10.1371/journal.pone.0163865 (PMC5055356; doi:10.1371/journal.pone.0163865)
Supplement: S1 Table — (DOC) [file pone.0163865.s001.doc]

**Supplementary Table 1 Hormone measurements in follicular and luteal phases of the same menstrual cycle.**

| **Hormones^a^** | **Follicular**  **(N=187)** | **Luteal**  **(N=187)** |
| --- | --- | --- |
| SHBG (nmol/L) | 54.9 (28.6) | 63.2 (34.0) |
| Oestradiol (pmol/L) | 194.0 (150.0) | 436.0 (251.0) |
| Free Oestradiol (pmol/L) | 2.7 (1.9) | 5.5 (2.9) |
| Progesterone (nmol/L) | 2.0 (1.0) | 24.0 (29.0) |
| Testosterone (nmol/L) | 1.7 (1.1) | 0.9 (0.7) |
| Free Testosterone (nmol/L) | 0.015 (0.010) | 0.007 (0.006) |
| Prolactin (ng/L) | 22.0 (16.0) | 15.0 (12.0) |

^a^ Hormone measurements are expressed as median (IQR)
