# Supplementary material for: Associations of Serum Levels of Sex Hormones in Follicular and Luteal Phases of the Menstrual Cycle with Breast Tissue Characteristics in Young Women
Source: PLoS One. 2016 Oct 7;11(10):e0163865. doi: 10.1371/journal.pone.0163865 (PMC5055356; doi:10.1371/journal.pone.0163865)
Supplement: S2 Table — N = 225. (DOC) [file pone.0163865.s002.doc]

**Supplementary Table 2: Simple and Multiple regression analysis of Water Volume^a^ (MRI) in young women (ages 15 – 30).N =225.**

| **Hormones** | ***Median, IQR***  *(*original and *log scale)* |  | ***Regression***  ***Coefficient***  *(adjusted ^b^)* |  | ***p-value***  *(adjusted)* | ***Median, IQR***  *(*original and *log scale)* |  | ***Regression***  ***Coefficient***  *(adjusted ^b^)* |  | ***p-value***  *(adjusted)* |
| --- | --- | --- | --- | --- | --- | --- | --- | --- | --- | --- |
|  |  |  |  |  |  |  |  |  |  |  |
| **SHBG^c^**  (nmol/L) | 54.5 [42.1; 69.5]  4.00 [3.74; 4.24] |  | *0.05 (0.12)* |  | **0.54 (0.12)** | 62.7 [44.9; 80.4]  4.14 [3.80; 4.39] |  | *0.04 (0.12)* |  | **0.62 (0.13)** |
| **Oestradiol^c^**  (pmol/L) | 187 [143; 286]  5.23 [4.96; 5.66] |  | *0.01 (0.05)* |  | **0.81 (0.43)** | 434 [329; 588]  6.07 [5.80; 6.38] |  | *-0.11 (-0.05)* |  | **0.13 (0.52)** |
| **FreeOestradiol^c^**  (pmol/L) | 2.68 [1.95; 3.76]  0.98 [0.67; 1.33] |  | *0.004 (0.017)* |  | **0.93 (0.79)** | 5.81 [4.35; 7.54]  1.76 [1.47; 2.02] |  | *-0.12 (-0.10)* |  | **0.09 (0.19)** |
| **Progesterone^c,d^**  (nmol/L) | 2.0 [2.0; 3.0]  0.69 [0.69; 1.10] |  | *-0.09 (-0.08)* |  | **0.16 (0.21)** | 23.0 [12.0; 41.0]  3.14 [2.48; 3.71] |  | *-0.07 (-0.04)* |  | **0.07 (0.40)** |
| **Testosterone**  (nmol/L) | 1.7 [1.3; 2.4] |  | *-0.06 (-0.06)* |  | **0.06 (0.08)** | 0.9 [0.6; 1.3] |  | *-0.03 (-0.04)* |  | **0.65 (0.57)** |
| **FreeTestosterone^c^**  (nmol/L) | 0.015 [0.01; 0.02]  -4.19 [-4.52; -3.82] |  | *-0.06 (-0.09)* |  | **0.22 (0.10)** | 0.0076 [0.0049; 0.0111]  -4.88 [-5.31; -4.50] |  | *-0.02 (-0.06)* |  | **0.65 (0.28)** |
| **Prolactin^c,e^**  (ng/L) | 22.0 [15.0; 32.0]  3.09 [2.71; 3.47] |  | *-0.08 (-0.06)* |  | **0.15 (0.28)** | 16.0 [10.0; 21.0]  2.77 [2.30; 3.04] |  | *-0.009 (-0.007)* |  | **0.88 (0.90)** |

^a^ Water volume was log transformed.

^b^ N = 224. Adjusted for: *Age at MRI* ; *Age at Menarche*; *Weight* ; *Height* and *Days since LMP* for each phase.

^c^ Log Transformed (regression coefficients were **not** back-transformed).

^d^ Progesterone values below the threshold were assigned the value of 1.

e N = 217 (N = 216 for the adjusted model) in the Follicular
