# Supplementary material for: Associations of Serum Levels of Sex Hormones in Follicular and Luteal Phases of the Menstrual Cycle with Breast Tissue Characteristics in Young Women
Source: PLoS One. 2016 Oct 7;11(10):e0163865. doi: 10.1371/journal.pone.0163865 (PMC5055356; doi:10.1371/journal.pone.0163865)
Supplement: S3 Table — N = 225. (DOC) [file pone.0163865.s003.doc]

|  | ***FOLLICULAR PHASE*** | | | | | ***LUTEAL PHASE*** | | | | |
| --- | --- | --- | --- | --- | --- | --- | --- | --- | --- | --- |
|  | ***Median, IQR***  *(*original and *log scale)* |  | ***Regression***  ***Coefficient***  *(adjusted ^b^)* |  | ***p-value***  *(adjusted)* | ***Median, IQR***  *(*original and *log scale)* |  | ***Regression***  ***Coefficient***  *(adjusted ^b^)* |  | ***p-value***  *(adjusted)* |
|  |  |  |  |  |  |  |  |  |  |  |
| **SHBG^c^**  (nmol/L) | 54.5 [42.1; 69.5]  4.00 [3.74; 4.24] |  | *-0.58(-0.22)* |  | **<0.0001 (0.013)** | 62.7 [44.9; 80.4]  4.14 [3.80; 4.39] |  | *-0.62(-0.24)* |  | **<0.0001 (0.009)** |
| **Oestradiol^c^**  (pmol/L) | 187 [143; 286]  5.23 [4.96; 5.66] |  | *-0.047 (-0.038)* |  | **0.57 (0.6)** | 434 [329; 588]  6.07 [5.80; 6.38] |  | *-0.11 (-0.006)* |  | **0.32 (0.95)** |
| **FreeOestradiol^c^**  (pmol/L) | 2.68 [1.95; 3.76]  0.98 [0.67; 1.33] |  | *0.1 (0.035)* |  | **0.22 (0.63)** | 5.81 [4.35; 7.54]  1.76 [1.47; 2.02] |  | *0.22 (0.11)* |  | **0.05 (0.19)** |
| **Progesterone^c,d^**  (nmol/L) | 2.0 [2.0; 3.0]  0.69 [0.69; 1.10] |  | *-0.12 (-0.12)* |  | **0.23 (0.11)** | 23.0 [12.0; 41.0]  3.14 [2.48; 3.71] |  | *-0.16 (-0.092)* |  | **0.012 (0.066)** |
| **Testosterone**  (nmol/L) | 1.7 [1.3; 2.4] |  | *-0.038 (-0.066)* |  | **0.44 (0.08)** | 0.9 [0.6; 1.3] |  | *0.11 (0.0045)* |  | **0.31 (0.95)** |
| **FreeTestosterone^c^**  (nmol/L) | 0.015 [0.01; 0.02]  -4.19 [-4.52; -3.82] |  | *0.22 (0.04)* |  | **0.0074 (0.53)** | 0.0076 [0.0049; 0.0111]  -4.88 [-5.31; -4.50] |  | *0.29 (0.08)* |  | **0.0001 (0.18)** |
| **Prolactin^c,e^**  (ng/L) | 22.0 [15.0; 32.0]  3.09 [2.71; 3.47] |  | *-0.054 (-0.041)* |  | **0.52 (0.52)** | 16.0 [10.0; 21.0]  2.77 [2.30; 3.04] |  | *0.077 (0.019)* |  | **0.41 (0.79)** |

**Supplementary Table 3: Simple and Multiple regression analysis of Fat Volume** ^a^ **(MRI) in young women (ages 15 – 30). N = 225.**

^a^ Fat volume was log transformed

^b^ N = 313. Adjusted for: *Age at MRI* ; *Age at Menarche*; *Weight* ; *Height* and *Days since LMP* for each phase.

^c^ Log Transformed (regression coefficients were **not** back-transformed)

^d^ Progesterone values below the threshold were assigned the value of 1.

^e^ N = 307 (N = 305 for the adjusted model) in the Follicular Ph
